# Rare but critical: Aberrant vascular communication leading to multiorgan ischemia after prophylactic gastroduodenal artery embolization for refractory upper gastrointestinal bleeding

**DOI:** 10.1016/j.radcr.2023.08.064

**Published:** 2023-08-27

**Authors:** Muhammad Ibrahim Saeed, Amna Subhan Butt, Jahanzeb Shahid, Junaid Iqbal

**Affiliations:** a Department of Medicine, Section of Gastroenterology, Aga Khan University Hospital, Karachi, Pakistan; b Department of Radiology, Aga Khan University Hospital, Karachi, Pakistan

**Keywords:** Refractory upper gastrointestinal bleeding, Prophylactic transcatheter arterial embolization, Vascular anatomic variation, Multiorgan ischemia

## Abstract

Upper gastrointestinal bleeding (UGIB) carries a high risk of morbidity and mortality despite recent improvements in diagnosis and management. Many patients failed to respond to initial endoscopic and medical management. Transcatheter arterial embolization (TAE) is now considered the next line of therapy over surgery in refractory UGIB because of its good safety profile and high technical and clinical success rate. We discuss the case of a 66-year-old female patient who presented with massive UGIB and had TAE after endoscopic hemostasis failed. She developed widespread ischemia involving multiple organs following the procedure, including the liver, gallbladder, pancreas, spleen, and small intestine, as a result of an abnormal communication between the gastroduodenal artery (GDA) and superior mesenteric artery (SMA), resulting in PVA particle reflux and widespread ischemic injury. It is important to carefully evaluate the vascular anatomic variations before the procedure to avoid potential complications of ischemia.

## Introduction

Upper gastrointestinal bleeding (UGIB) is an acute medical emergency with an estimated mortality of around 10% [1,2]. Despite improvements in diagnosis and management over the last 50 years, mortality stayed high if remain untreated [1–3]. After initial resuscitation early endoscopic intervention is highly effective first-line therapy for achieving hemostasis in the majority of patients; nevertheless, many patients rebleed after successful initial hemostasis [4]. Transcatheter arterial embolization (TAE) has replaced surgery as the first-line therapy in patients with nonvariceal UGIB who have failed to respond to endoscopic procedures due to its high technical and clinical success and good safety profile rates [5,6]. We report the case of a 66-year-old female patient who presented with massive UGIB and had TAE after endoscopic hemostasis failed. She developed widespread multiorgan ischemia following the procedure.

## Case summary

**Fig. 1:**
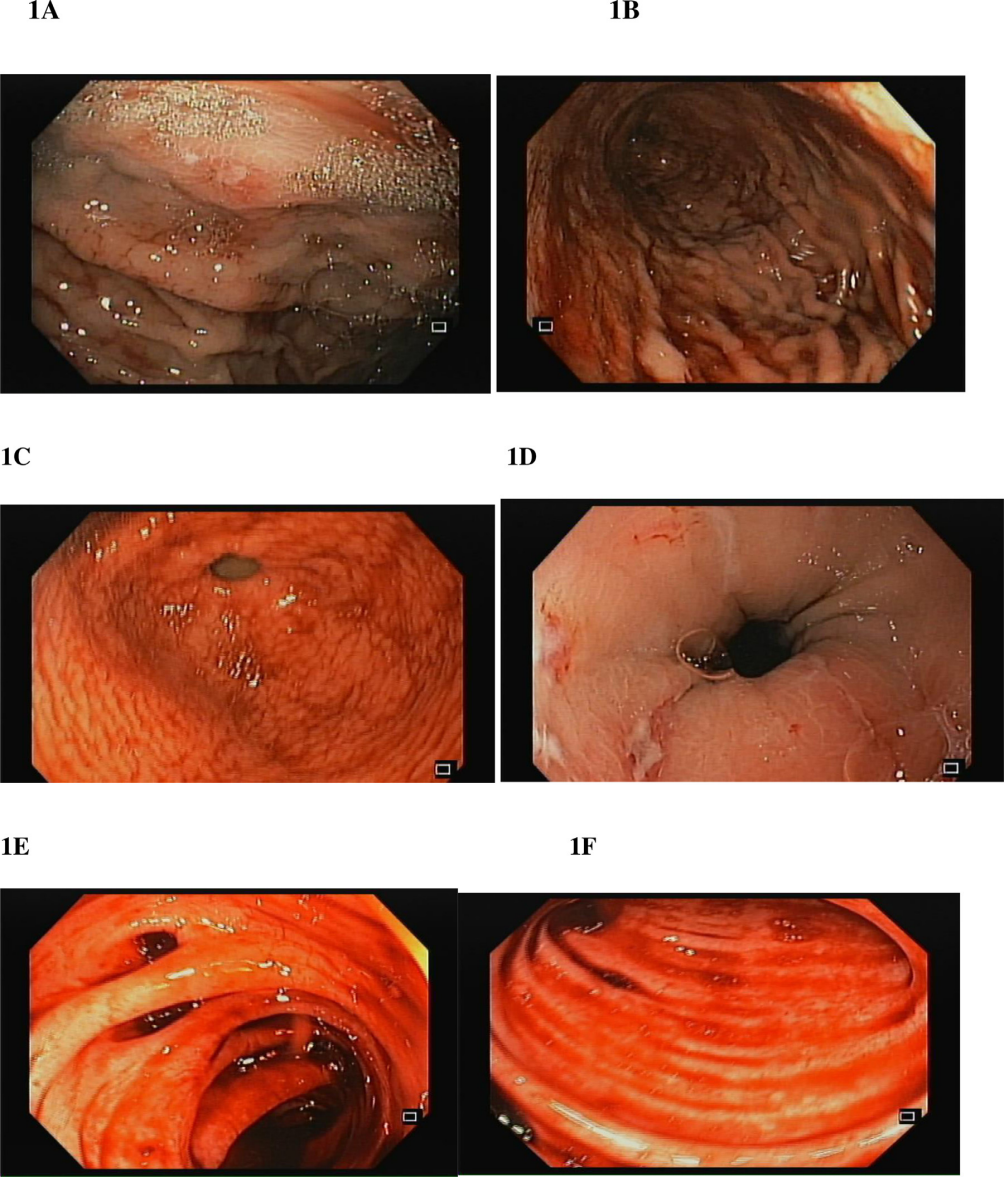
Images from the first endoscopy: (A) Multiple erosions and small ulcers in gastric body, (B) gastric mucosa smeared with blood, (C) antral mucosa smeared with blood, (D) ulcers below Z line after adrenaline sclerotherapy, (E) ascending colon smeared with blood, (F) sigmoid colon with multiple diverticula, and blood-stained mucosa.

**Fig. 2:**
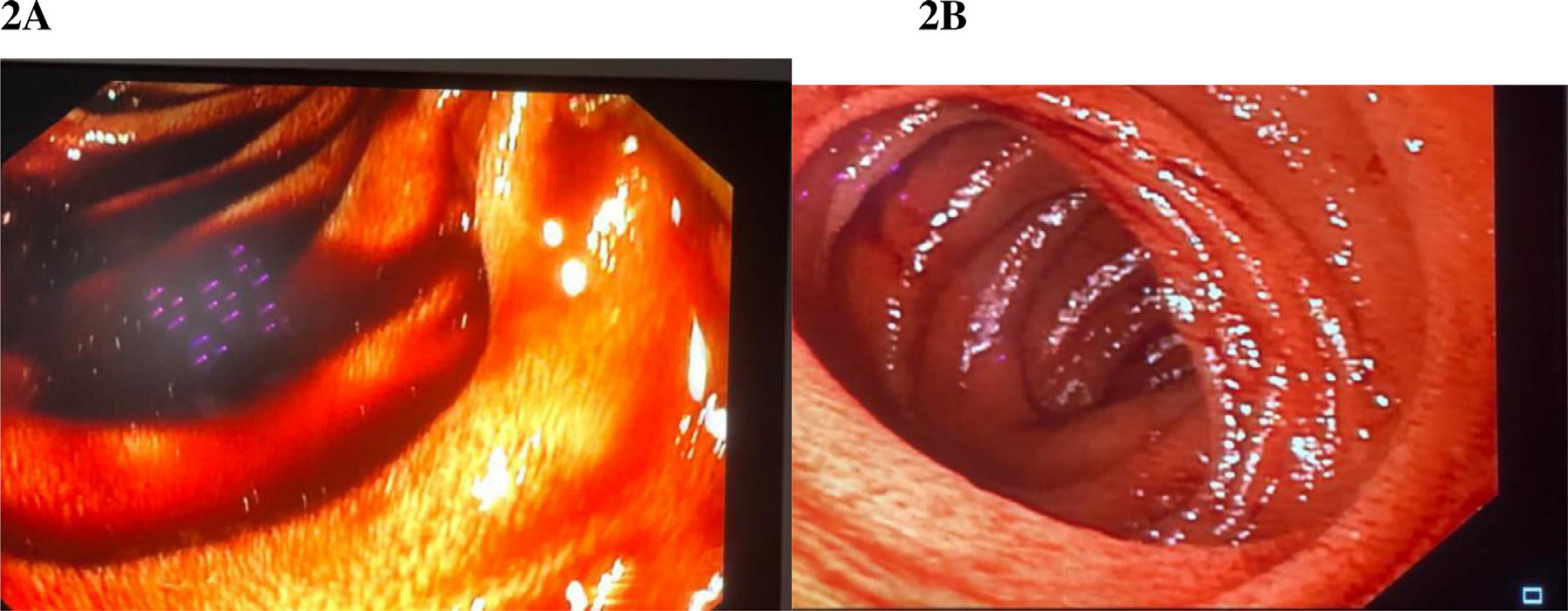
Images from enteroscopy: (A) showing active pooling of blood in the third part of the duodenum, (B) Jejunum blood smeared mucosa.

**Fig. 3:**
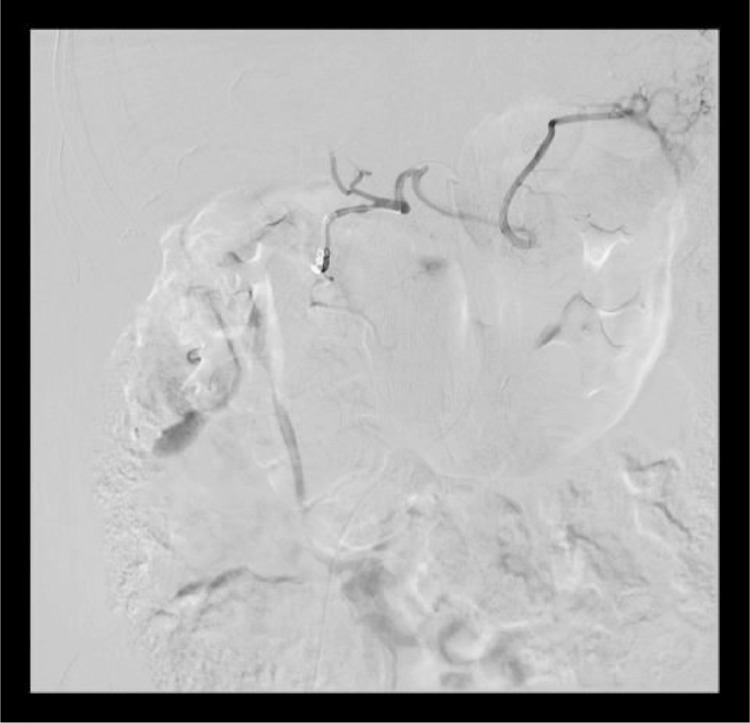
DSA of celiac artery showing coil embolization of gastroduodenal artery (Note that the common origin of the celiac and SMA are not readily visible on DSA).

**Fig. 4:**
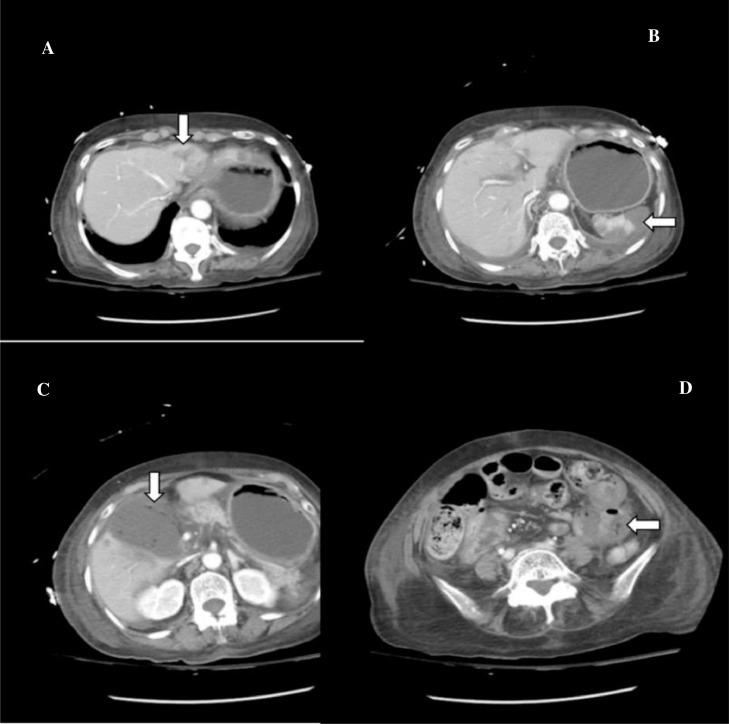
Multiple axial images from contrast-enhanced abdominal CT. (A) shows a low attenuation area in the liver representing liver infarction (white arrow). (B) shows multiple splenic infarctions (white arrow). (C) shows air specks in the wall of the gall bladder secondary to ischemia (white arrow). (D) shows the absent enhancement of small bowel loops secondary to mesenteric ischemia (white arrow).

**Fig. 5:**
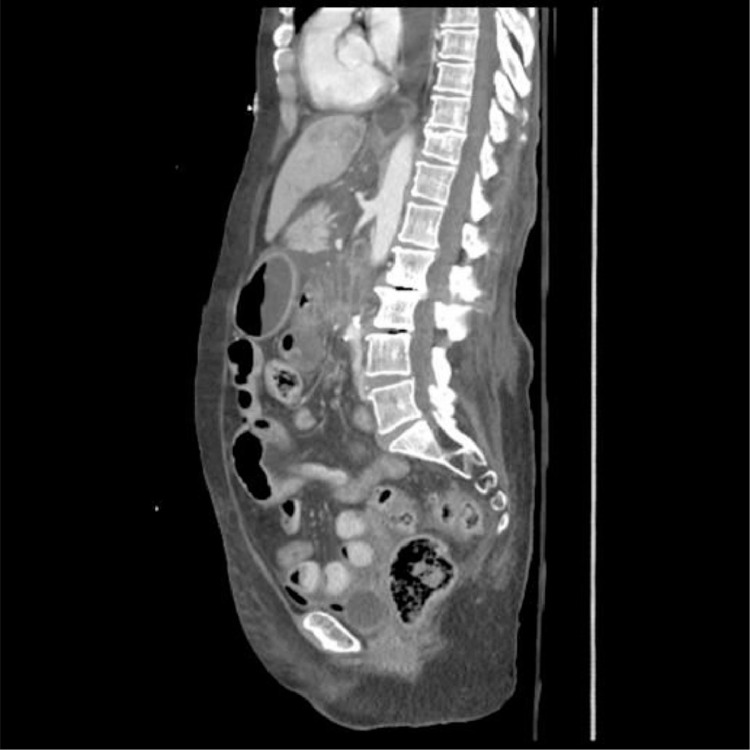
Sagittal image of contrast-enhanced abdominal CT, showing the common origin of the celiac and superior mesenteric artery.

A 66-year-old South Asian woman with a history of hypertension, systemic lupus erythematosus, and asthma presented to the emergency department with coffee-brown emesis, melena, and bleeding per rectum for the past 3 days. She was using NSAIDs for the pain in her joints. She received 2 units of packed red blood cells (RBCs) in a local hospital the day before arriving at the emergency room since her hemoglobin (Hb) was 30 g/L (normal range in females 121-151 g/L). On arrival, her heart rate (HR) was 139 beats per minute with a blood pressure of 100/50, while maintaining oxygen saturation on room air. On examination, pallor, mild tenderness in the upper abdomen, and melanotic stools on digital rectal examination were noticed. Her laboratory parameters in ER revealed Hb of 39 g/L, WBC 5.8×10^9^/L (normal range 4-11×10^9^/L), and platelet counts 184×10^9^/L (normal range: 150-350×10^9^/L). Her prothrombin time, creatinine, and electrolytes were all within the normal limits. After resuscitation with intravenous fluid, intravenous omeprazole, and transfusing 3 more packed red blood cells an esophagogastroduodenoscopy (EGD) was performed which revealed multiple erosions and a few tiny ulcers scattered throughout the gastric body, a hiatal hernia and oozing from 1 ulcer below the Z line that settled after adrenaline sclerotherapy (Figs. 1A–D). Her Hb was 67 g/L after EGD, hence 2 more packed RBCs were transfused. She didn't experience any episodes of overt bleeding following EGD, but since her Hb was 87g/L, 2 more packed RBCs were transfused. The next morning at 4 am, she passed 2 stools with a large quantity of maroonish blood, became tachycardic (HR 120-125 beats/min), and her hemoglobin level dropped to 64 g/L. Two packed RBCs were transfused. Relook EGD confirmed that all previously identified ulcers were in the healing phase, without any stigmata of recent or active bleeding. Consequently, a colonoscopy was performed, which revealed blood-smeared mucosa up to the cecum as well as several diverticula in the sigmoid colon but no active bleeding spots were identified (Figs. 1E and F). A contrast-enhanced computed tomography scan (CT) with CT angiography was done, and no active extravasation of high-density contrast was found to suggest active bleeding. She remained stable till she had 2 episodes of maroonish blood in her stool on day 6, became tachycardic, and her Hb dropped to 61 g/L. Following resuscitation with packed RBCs and intravenous hydration, an urgent enteroscopy was performed by a second endoscopist to evaluate the small bowel as a potential source of bleeding, which revealed a pool of blood in the third part of the duodenum (Figs. 2A and B). Despite vigorous flushing and suctioning the bleeding spot was not apparent. There were no signs of an active or recent bleed in the stomach or remaining small bowel. A transarterial angiography of the celiac and mesenteric arteries was performed, demonstrating spasm and aberrant blush at the gastroduodenal artery (GDA), but no contrast extravasation was detected. Considering the fact that the persistent bleeding required 15 units of packed RBC transfusion so far, a pool of fresh blood in the third part of the duodenum, and aberrant blush at GDA suggesting a bleed in the small bowel, it was decided to proceed with empiric GDA embolization. The GDA was embolized using two 3×50 mm coils and 250-355 polyvinyl alcohol (PVA) particles (Fig. 3). There was no aberrant flush after the embolization. The patient complained of mild abdomen pain that eventually became severe a few hours after the procedure was performed. Her laboratory parameters revealed Hb 119 g/L, total leucocyte counts 19.5×10^9^/L, serum lactate 6.5 mmol/L (normal < 1 mmol/L), amylase 428 IU/L (40-140 U/L), total bilirubin of 1.2 mg/dL (normal < 1 mg dL), GGT 25 IU/L(normal: 5-40 IU/L), ALT 514 IU/L(normal: 4-35 IU/L), AST 567 IU/L(normal: 8-33 IU/L), and Alkaline phosphatase 44 IU/L(normal: 44-147 IU/L). A contrast-enhanced CT scan of the abdomen was carried out immediately (Figs. 4A–D) which showed multiple infarcts in the liver, spleen, gall bladder, head of the pancreas, and mesenteric ischemia involving gastric pylorus, duodenum, and the proximal jejunum as a result of an abnormal communication between the GDA and superior mesenteric artery (SMA) (Fig. 5), resulting in PVA particle reflux and widespread ischemic injury. A multidisciplinary team comprising a vascular surgeon, hepatobiliary surgeon, and intervention radiologist was involved in exploring possible treatment options. Surgery was not possible since the ischemia was widespread and multiple organs were affected. Another option was to give local thrombolytic agents, but there was no celiac or other major vessel thrombosis on the CT exam. She was started on heparin infusion and shifted to the intensive care unit for further management. Later, she was intubated because of respiratory distress and worsening lactic acidosis. Post TAE she didn't have a further episode of bleed and Hb remained stable, however, she expired after 3 days of TAE as a consequence of widespread ischemia to multiple organs.

## Discussion

Prophylactic transcatheter arterial embolization (TAE) refers to performing embolization in cases where there is no extravasation of contrast at the time of an angiogram or CT scan performed with GI bleeding protocol. This is considered in patients with a high risk of rebleeding, such as Forrest class I-IIb ulcers, large ulcers, individuals with hemodynamic instability, etc., and those with low pressures during endoscopy interventions [7,8]. It is highly effective in reducing the risk of rebleeding and carries a technical success rate of up to 100% and a clinical success rate of around 79% with a good safety profile [5,6]. Common complications include rebleeding and access site-related complications such as hematoma and pseudoaneurysm formation. Other rare adverse events are arterial dissection, coil migration, and anaphylaxis [8,9].

Ischemic complications affecting the upper gastrointestinal tract have been reported in up to 7%-16% of cases despite rich anastomosis between the main arterial supply in the stomach and duodenum [10]. The risk increases with prior history of surgery in the gastrointestinal tract or with the type of embolic agents used, such as liquid agents (eg, cyanoacrylate) or tiny particles (gelatin sponge or microspheres) that can spread far into the vascular circulation [7]. Some studies have reported up to 2.7% of ischemic events in the acute phase and 25% of duodenal stenosis due to chronic ischemia [11,12].

The gastroduodenal artery (GDA) arises from the common hepatic artery; the variable origins include the superior mesenteric artery, the left hepatic artery, and the proper hepatic artery [13]. It supplies the gastric antrum, proximal duodenum, and the head of the pancreas. Anatomic variations in the celiac artery occur in approximately 50% of the population, making it an important consideration when evaluating patients for TAE [14]. The duodenum receives blood from the celiac and the superior mesenteric artery (through the inferior pancreatic duodenal arcades); therefore, the culprit vessel should be embolized for adequate hemostasis proximal and distal to the bleeding point [15]. This method is known as the sandwich technique, where both ends of the GDA are filled with coils to block retrograde bleeding from the SMA. This also prevents the distal spread of micro embolic particles, especially when there is an aberrant communication, and hence minimizes the chances of ischemia.

In our case, abnormal communication between GDA and SMA was not picked up initially on DSA because of severe vascular spasms. With the perception of no aberrant communication in mind, initially, PVA particle embolization of GDA was done, followed by proximal coil embolization of GDA at its origin instead of the sandwich technique of embolization. This later resulted in small bowel ischemia as PVA particles refluxed via aberrant communication to SMA. After retrospective evaluation of pre-embolization CT angiogram, one additional finding that was noted was the common origin of the celiac artery and SMA. This may also have contributed to the reflux of PVA particles from celiac artery distribution to SMA territory, the main reason for widespread ischemia.

## Conclusion

Prophylactic transcatheter arterial embolization of GDA has replaced surgery because of its good safety profile and excellent technical and clinical success rate in patients with refractory UGIB. Anatomic variations exist and images should be carefully evaluated before the procedure to avoid potential complications of ischemia.

## Statement of ethics

Ethical approval was granted by the Ethics Review Committee.

## Authors contribution

Amna Subhan and Ibrahim Saeed wrote the original draft, Jahanzeb, and Junaid collected and Interpreted the imaging. All authors have read, revised, and approved the final published version of the manuscript. All authors were responsible for submission of our study for publication.

## Patient consent

Written informed consent was obtained from the patient's husband for the publication of this case report and accompanying images.
